# Association of sarcopenia and muscle attenuation with clinical outcomes in patients with diabetic foot: a systematic review and meta-analysis

**DOI:** 10.3389/fendo.2026.1894356

**Published:** 2026-08-21

**Authors:** Li Yang, Lei Wang, Jiaqi Yang, Yang Li, Xuan Li, Bing Wu, Ting Yin, Yi Zhao

**Affiliations:** 1 Department of Epidemiology and Health Statistics, School of Public Health, Ningxia Medical University, Yinchuan, Ningxia, China; 2 Department of Rehabilitation Medicine, General Hospital of Ningxia Medical University, Yinchuan, Ningxia, China; 3 Department of Oral Pathology, College of Stomatology, Ningxia Medical University, Yinchuan, Ningxia, China; 4 Department of Traditional Chinese Medicine Rehabilitation, Ningxia Disabled Persons’ Rehabilitation Center (Ningxia Rehabilitation Hospital), Yinchuan, Ningxia, China; 5 Ningxia Key Laboratory of Environmental Factors and Chronic Disease Control, Ningxia Medical University, Yinchuan, Ningxia, China

**Keywords:** amputation, clinical prognosis, diabetic foot, meta-analysis, mortality rate, sarcopenia and muscle attenuation, systematic review, ulcer severity

## Abstract

**Background:**

Diabetic foot (DF) represents a severe and disabling complication of diabetes, closely linked to ulceration, amputation, functional impairment, and an elevated risk of death. Sarcopenia is a syndrome marked by progressive declines in muscle mass and function. Emerging evidence suggests a possible link between DF and sarcopenia. Nonetheless, a systematic evaluation of the influence of sarcopenia on adverse clinical outcomes in individuals with DF remains unavailable. The present investigation aims to systematically assess and quantitatively synthesize the association of sarcopenia and muscle attenuation with clinical outcomes in those with DF.

**Methods:**

A systematic search of Embase, Cochrane, PubMed, Web of Science, SinoMed, China National Knowledge Infrastructure, Wanfang, and VIP databases was conducted from their inception through December 2025. Observational studies enrolling DF individuals aged ≥ 18 years and comparing clinical prognoses between those with and without sarcopenia and muscle attenuation were included. The primary outcomes comprised rates of mortality and amputation. Secondary outcomes encompassed ulcer severity. The NHLBI was adopted for risk-of-bias evaluation. Given substantial between-study heterogeneity, a narrative synthesis was prioritized, with meta-analyses implemented for outcomes amenable to quantitative pooling.

**Results:**

Eighteen studies (9 cross-sectional, 4 prospective cohort, 4 retrospective cohort, and 1 retrospective case-control studies) comprising 2,888 individuals were included, of which 9 contributed to the meta-analysis. The meta-analysis revealed that, relative to those without sarcopenia and muscle attenuation, DF individuals with comorbid sarcopenia exhibited an elevated risk of all-cause mortality (HR = 2.38, 95% CI: 1.63–3.48), an association with amputation (OR = 2.11, 95% CI: 1.32–3.38), and a higher proportion of severe ulcers (Wagner grade 4–5) (RR = 1.69, 95% CI: 1.31–2.17).

**Conclusions:**

Impaired muscle health was associated with a higher risk of all-cause mortality, higher odds of amputation, and a greater prevalence of severe ulcers in patients with DF. Because the available evidence is observational and heterogeneous, these findings should be considered hypothesis-generating and should not yet be used to support prognostic screening or clinical risk-stratification decisions. Future prospective cohort investigations are necessary to further clarify the temporal relationship and underlying mechanisms.

**Funding and registration:**

This study was supported by the Ningxia Natural Science Foundation (Grant Number: 2025AAC030725). This systematic review complied with the Preferred Reporting Items for Systematic Reviews and Meta-Analyses (PRISMA) guidelines and was registered in the International Prospective Register of Systematic Reviews (PROSPERO: CRD420251247873).

**Systematic Review Registration:**

https://www.crd.york.ac.uk/prospero/, identifier CRD420251247873.

## 1 Introduction

Diabetic foot (DF) constitutes a severe and disabling complication of diabetes and a principal contributor to heightened risks of lower-extremity amputation and death in diabetic populations (1). Diabetic foot ulcers (DFU) represent among the most serious clinical manifestations of long-term diabetic complications, with an estimated global prevalence of around 6.3% (95% confidence interval [CI]: 5.4%-7.3%) (2). Their occurrence and progression are closely linked to multiple factors, including neuropathy, peripheral vascular disease, infection, and repetitive trauma (3). Owing to protracted disease courses, recurrent wound infections, diminished mobility, and enduring functional impairment, DF inflicts local tissue damage and is associated with severe ulcers, amputation, death, and deteriorated quality of life (4). Furthermore, DF imposes a considerable economic burden on patients’ families and healthcare systems, and its treatment costs engender substantial social and familial strain, particularly in resource-limited settings (5).

Sarcopenia is a progressive, generalized skeletal muscle disorder characterized by the loss of muscle mass, strength, and physical performance (6, 7). According to the European Working Group on Sarcopenia in Older People (EWGSOP2) consensus, the diagnosis of sarcopenia is based on three core domains: low muscle strength (the key defining characteristic), low muscle quantity or quality, and low physical performance (8). The Asian Working Group for Sarcopenia (AWGS) 2019 consensus similarly defines sarcopenia as age-related loss of muscle mass, plus low muscle strength and/or low physical performance (9). In clinical practice and research, sarcopenia is commonly identified using handgrip strength (HGS), appendicular skeletal muscle mass index (assessed by bioelectrical impedance analysis or dual-energy X-ray absorptiometry), and gait speed or other physical performance tests. This syndrome may substantially elevate the risk of falls, fractures, functional decline, disability, and death. Among diabetic individuals, sarcopenia correlates not only with reduced mobility and impaired quality of life but also likely with heightened risks of fractures, functional deterioration, and death (10). Prior research indicates that sarcopenia is likewise related to worse prognoses in diabetic individuals undergoing amputation (11). Hence, sarcopenia and muscle attenuation may represent more than a mere concomitant condition, functioning instead as a clinically relevant factor influencing the trajectory of chronic diabetic complications.

Recently, growing evidence has pointed toward a possible close relationship of DF with sarcopenia and muscle attenuation. Available data suggest that indicators such as sarcopenia, low HGS, low skeletal muscle index, low muscle mass, or low muscle density may be associated with heightened risk of death, elevated risk of amputation, aggravated severity of ulcers, and poor wound healing in individuals with DF (12–16). HGS, a critical index of muscle function, has also been suggested to be linked to healing rates and risk of amputation in this population (16, 17). Nonetheless, findings across studies remain inconsistent. For instance, some investigations report that low HGS may correlate with an elevated risk of limb loss, whereas others detect no significant difference (12). Outcomes concerning peripheral neuropathy prove similarly discordant. Certain studies suggest that it is not independently related to amputation, while others indicate that the severity of neuropathic symptoms may correlate with sarcopenia (18). Such inconsistencies possibly stem from variations in study design, sample size, diagnostic criteria of sarcopenia and muscle attenuation, selection of indicators for muscle attenuation, and outcome definitions.

The relationship of sarcopenia with clinical outcomes—including death, amputation, ulcer severity, healing, and peripheral neuropathy—in individuals with DF remains unsettled. Most existing studies are single-center observational investigations with limited sample sizes and appreciable heterogeneity in design and outcome definitions. Consequently, a systematic evaluation and meta-analysis addressing the link between sarcopenia and clinical outcomes in individuals with DF is currently lacking. Accordingly, the present investigation, through systematic review and meta-analysis, aims to comprehensively examine the associations of sarcopenia and muscle attenuation and abnormal objective indicators for muscle attenuation with death, amputation, ulcer severity, and other pertinent clinical outcomes in these individuals, thereby clarifying the current evidence base and identifying priorities for future prognostic and interventional research.

## 2 Methods

This systematic review complied with the Preferred Reporting Items for Systematic Reviews and Meta-Analyses (PRISMA) guidelines (19) and received registration in the International Prospective Register of Systematic Reviews (CRD420251247873). A complete PRISMA 2020 checklist is available in **Supplementary Table 1**.

### 2.1 Data sources

A systematic search of Embase, Cochrane, PubMed, Web of Science, SinoMed, China National Knowledge Infrastructure (CNKI), Wanfang, and VIP databases was executed for relevant studies up to December 3, 2025. A search strategy combining free-text and controlled vocabularies was adopted, with search syntax constructed around terms related to ‘diabetic foot’, ‘diabetic feet’, ‘diabetic foot disease’, and ‘sarcopenia’. No language restrictions were imposed. Detailed search procedures are available in **Supplementary Table 2**.

### 2.2 Study selection

Inclusion criteria were formulated utilizing the PECOS framework. Population (P): individuals with DF aged ≥ 18 years; Exposure (E): sarcopenia and muscle attenuation (diagnosed as ‘sarcopenia’ per international consensus criteria, or studies reporting ‘low muscle mass’, ‘low skeletal muscle index’, ‘muscle attenuation’, or ‘low muscle density’ with grouping based on objective muscle indicators); Comparator (C): DF individuals without sarcopenia and low muscle attenuation; Outcomes (O): primary outcomes encompassing rates of mortality and amputation; secondary outcomes including ulcer severity, wound healing, peripheral neuropathy, polyvascular disease, chronic kidney disease, malnutrition, and wound pH; Study design (S): observational investigations, comprising cross-sectional studies and prospective/retrospective cohort studies.

Exclusion criteria: (i) duplicate publications; (ii) studies with incomplete data or from which requisite outcome measures could not be extracted; (iii) basic experimental investigations and animal studies; (iv) letters, conference abstracts, reviews, and dissertations.

### 2.3 Literature screening

A four-stage strategy was applied. First, EndNote X21 was adopted to remove duplicates by title. Second, two independent reviewers (Li Yang and Lei Wang) separately screened the titles and abstracts to evaluate relevance. Third, studies from the prior step were examined in full text to determine their eligibility for quantitative synthesis. Ultimately, a third reviewer (Yang Li) audited and aggregated screening results, with disagreements addressed through discussion.

### 2.4 Risk-of-bias assessment

Two independent reviewers (Li Yang, Lei Wang) conducted risk-of-bias evaluation for eligible studies per prespecified criteria. A third reviewer (Yang Li) audited and aggregated the assessments. Discrepancies were settled through discussion. For cross-sectional and cohort studies, the National Heart, Lung, and Blood Institute Quality Assessment Tool for Observational Cohort and Cross-Sectional Studies was employed. This instrument comprises 14 items covering 6 core dimensions: research aim, study population and sample size, exposure measurement, outcome measurement, completeness of follow-up, and confounding bias control. Each item offers 5 rating options: ‘Yes’, ‘No’, ‘Cannot determine’, ‘Not applicable’, and ‘Not reported’. Overall quality of the eligible studies was classified per total scores as high (good), moderate (fair), and low (poor).

### 2.5 Data extraction

Two independent reviewers extracted data using a predesigned standardized form and cross-checked their results. Discrepancies were resolved through discussion or adjudicated by a third reviewer when necessary. Collected information encompassed: first author, publication year, country, study design, sample size, demographics (sex composition, age, body mass index [BMI]), and diabetes duration. Outcome data comprised mortality rate, amputation rate, ulcer severity, wound healing, peripheral neuropathy, polyvascular disease, malnutrition, and wound pH. For each outcome, we extracted the effect estimate (crude or adjusted), its 95% confidence interval, and the adjustment variables when applicable. When multiple adjustment models were reported, the model with the most comprehensive adjustment for potential confounders was prioritized. When multiple time points were reported, the longest available follow-up data were extracted. Outcome definitions were recorded as reported in the original studies.

### 2.6 Data synthesis

Given marked heterogeneity across the eligible studies in research design, exposure definitions, and outcome measurements, a narrative synthesis was prioritized, and meta-analyses were carried out solely for outcomes exhibiting adequate comparability. For outcomes with relatively consistent definitions, comparable reporting formats, and extractable raw data, quantitative pooling was further executed.

Effect measures were selected according to outcome type and data availability. All-cause mortality was synthesized using adjusted hazard ratios (HRs). Amputation was synthesized using adjusted odds ratios (ORs). Severe ulcer severity was synthesized using crude risk ratios (RRs) calculated from raw event counts. The pooled estimates for ulcer severity were derived from unadjusted data, as the adjusted estimates reported in the primary studies varied considerably in their adjustment sets and outcome definitions, precluding meaningful pooling of adjusted effect estimates for this outcome. In contrast, the pooled estimates for mortality and amputation were based on adjusted effect sizes. Between-study heterogeneity was evaluated via Cochran’s Q test and the I² statistic (20, 21). When I² > 50% accompanied by P < 0.05, indicating substantial heterogeneity, a random-effects model (DerSimonian-Laird approach) was applied; when I² ≤ 50% and P ≥ 0.05, a fixed-effects model was employed. To assess the robustness of the findings, sensitivity analyses were implemented by iteratively removing individual studies and repeating the meta-analysis to examine the influence of single investigations on overall estimates. Publication bias was assessed using funnel plots. When the number of eligible studies for a single outcome was ≥ 10, Egger’s regression test was additionally applied to evaluate publication bias. Meta-regression was prespecified but was not performed because fewer than 10 studies were available for each outcome. Statistical analyses were completed in R (v4.5.2).

## 3 Results

### 3.1 Study selection

**Figure 1:**
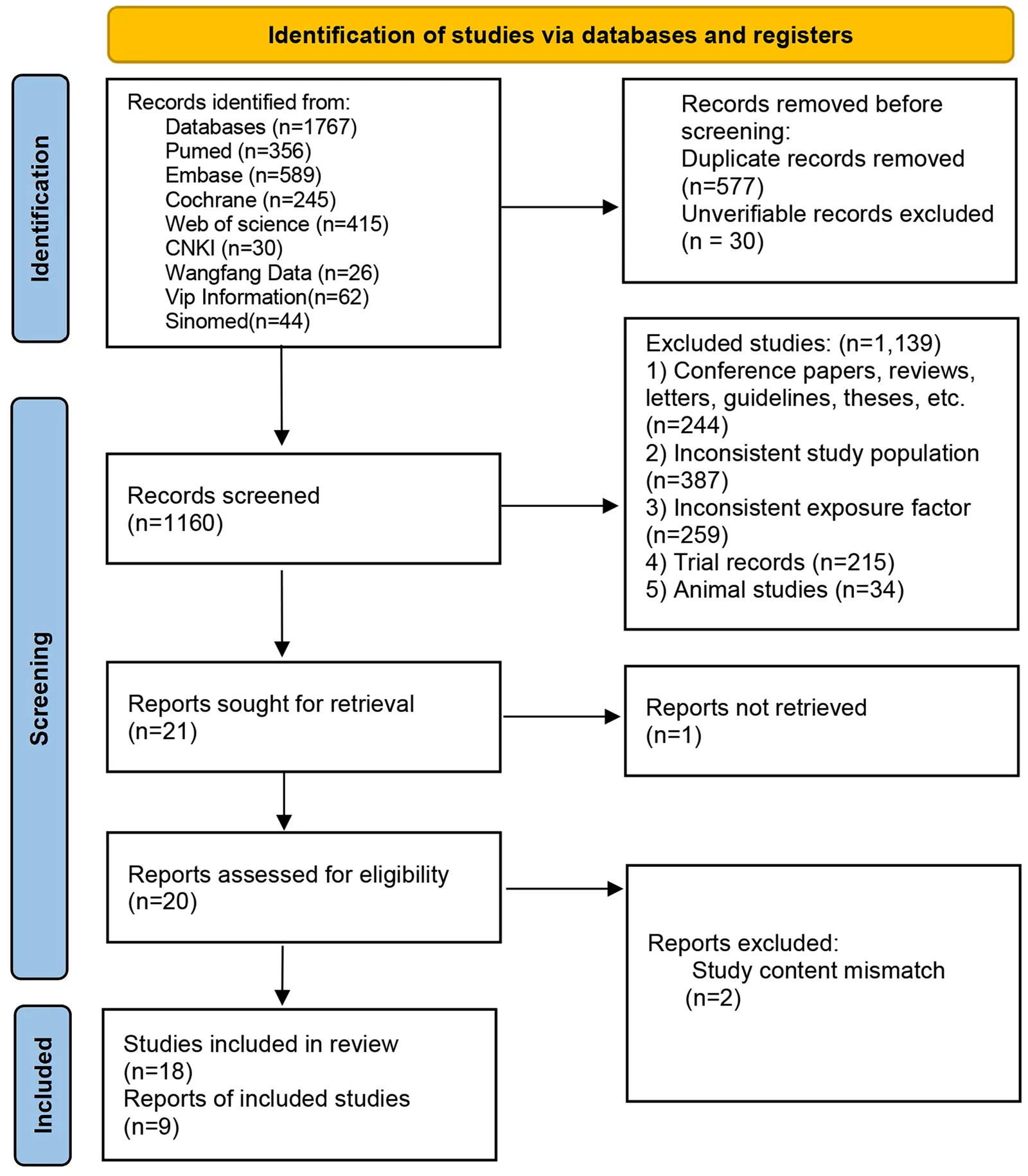
PRISMA flow diagram for the screening of studies.

The initial retrieval produced 1,767 records. After removing 577 duplicates and 30 unverifiable records, 1,160 records were reviewed by title and abstract. Of these, 1,139 were eliminated, and 21 records were reviewed in full text. Of the 21 reports sought for full-text review, one could not be retrieved. Among the 20 reports assessed for eligibility, two were excluded due to content mismatch. Ultimately, 18 studies were incorporated in the systematic review and 9 in the meta-analysis. The screening process is illustrated in the PRISMA flow diagram (**Figure 1**).

### 3.2 Baseline characteristics and risk-of-bias assessment

#### 3.2.1 Baseline characteristics

Eighteen studies were included: 9 cross-sectional, 4 prospective cohort, 4 retrospective cohort, and 1 retrospective case-control. The total sample comprised 2,888 participants (case group: n = 908; control group: n = 1,980). Study populations originated from multiple countries and regions, including China, Korea, Turkey, Spain, and Australia. General demographic characteristics of the 9 studies eligible for the meta-analysis are detailed in **Table 1**. These studies contributed to the quantitative syntheses, with two studies contributing to more than one outcome. The study contributions to each meta-analysis are detailed in **Supplementary Table 4**.

**Table 1:** Baseline characteristics of the eligible studies.

First author	Publication year	Study design	Country	Group	Sample size	Age	Sex ratio (M%)	Body mass index	Diabetes duration (years)	Outcomes	Effect measure
An	2024	Cross-sectional	China	DF+ Sarcopenia	44	67.27 ± 7.93*	77.30%	21.80 ± 2.45	18.5 ± 7.41*	Ulcer severity	crude
				DF without Sarcopenia	64	61.09 ± 9.43	59.40%	24.57 ± 3.21	10 ± 10*		
Valverde	2023	Prospective Cohort	Spain	DF+ Sarcopenia	41	73.7 ± 9.5	75.61%	26.3 ± 4.3	21.9 ± 10.2	Amputation rate, Mortality rate,	Amputation rate: crude, Mortality rate: adjusted
				DF without Sarcopenia	36	64.8 ± 11.8	76.47%	29.4 ± 6.4	20 ± 10.9		
Kim	2018	Retrospective Cohort	Korea	DF+ Sarcopenia	112	63.1 ± 11.6	80.36%	21.1 ± 3.2	**—**	Mortality rate	adjusted
				DF without Sarcopenia	55	60.5 ± 11.5	50.91%	24.3 ± 3.1	**—**		
Yang	2023	Prospective Cohort	China	DFU+ Sarcopenia	81	69.86 ± 11.23	80.25%	**—**	**—**	Mortality rate	adjusted
				DFU without Sarcopenia	110	65.39 ± 10.73	60.00%	**—**	**—**		
Cheng	2017	Cross-sectional	China	DF+ Sarcopenia	43	**—**	**—**	**—**	**—**	Amputation rate, ulcer severity	Amputation rate: adjusted, ulcer severity: crude
				DF without Sarcopenia	77	**—**	**—**	**—**	**—**		
Hon	2025	Prospective Cohort	Australia	DF+ Sarcopenia	16	72 ± 11.85*	31.25%	**—**	21 ± 14.07*	Mortality rate	Mortality rate: adjusted
				DF without Sarcopenia	84	71 ± 11.85*	76.19%	**—**	13 ± 7.78*		
IMRE	2022	Cross-sectional	Turkey	DFU+ Sarcopenia	49	70 ± 8.15*	44.90%	26.42 ± 2.99*	20 ± 10.37*	Ulcer severity	crude
				DFU without Sarcopenia	40	75 ± 8.52*	55.00%	35.11 ± 8.83*	15 ± 7.41*		
IMRE	2024	Cross-sectional	Turkey	DFU+ Sarcopenia	99	57 ± 6.67*	31.31%	29.29 ± 5.88*	12 ± 7.63*	Ulcer severity	crude
				DFU without Sarcopenia	143	55.5 ± 6.89*	32.87%	29.70 ± 4.52*	15 ± 6.30*		
IMRE	2024	Prospective Cohort	Turkey	DFU+ Sarcopenia	100	62.06 ± 9.01	35.00%	29.21 ± 5.04*	13 ± 7.78*	Amputation rate, ulcer severity	Amputation rate: adjusted, ulcer severity: crude
				DFU without Sarcopenia	105	58.05 ± 9.51	36.05%	31.22 ± 7.09*	15 ± 6.67*		

DF, diabetic foot; DFU, diabetic foot ulcers; M, male; BMI, body mass index; NR, not reported. Data are presented as mean ± SD as reported in the original studies. Values marked with an asterisk (*) were estimated from the reported median and interquartile range (IQR) using the formula proposed by Wan et al. (SD ≈ IQR/1.35) when means and SDs were not directly reported. These estimated values are clearly distinguished from directly reported means to facilitate appropriate interpretation. “—” indicates data not reported in the original study.

Among the 18 investigations, sarcopenia was defined using consensus criteria in 2 studies, isolated low muscle strength in 5, isolated low muscle quantity/mass in 6, a biochemical surrogate index in 1, non-consensus composite or mixed criteria in 3, and an imaging-derived measure without a diagnostic cutoff in 1. Given the heterogeneity of exposure definitions across studies, “sarcopenia and muscle attenuation” was used as an umbrella term for impaired muscle health. Detailed exposure classifications are provided in **Supplementary Table 3**; therefore, the pooled estimates should be interpreted as associations with impaired muscle health rather than effects of uniformly diagnosed sarcopenia.

#### 3.2.2 Risk-of-bias assessment

Eighteen publications (11–18, 22–31) underwent quality appraisal, comprising 9 cross-sectional, 4 prospective cohort, 4 retrospective cohort, and 1 retrospective case-control studies. Of these, 7 were rated as good quality and 11 as fair quality. Details are available in **Supplementary Table 5**.

These eligible studies exhibited an overall moderate risk of bias, attributable mainly to the following: (i) a cross-sectional design of 9 investigations (11, 22, 23, 25–27, 29–31), which constrained causal inference; (ii) issues of sample representativeness in 9 investigations (11, 15, 22, 24, 25, 28–31), with certain studies having relatively small sample sizes or selection bias; (iii) insufficient confounding control in 2 investigations (15, 24), with some possible confounders inadequately measured or adjusted; (iv) measurement bias in 3 studies (15, 22, 24), owing to suboptimal standardization in measurement approaches for certain exposures or outcomes. These quality limitations were carefully considered during the analysis of data and interpretation of results.

### 3.3 Meta-analysis results

#### 3.3.1 Primary outcomes

##### 3.3.1.1 Sarcopenia and Mortality Rate

**Figure 2:**
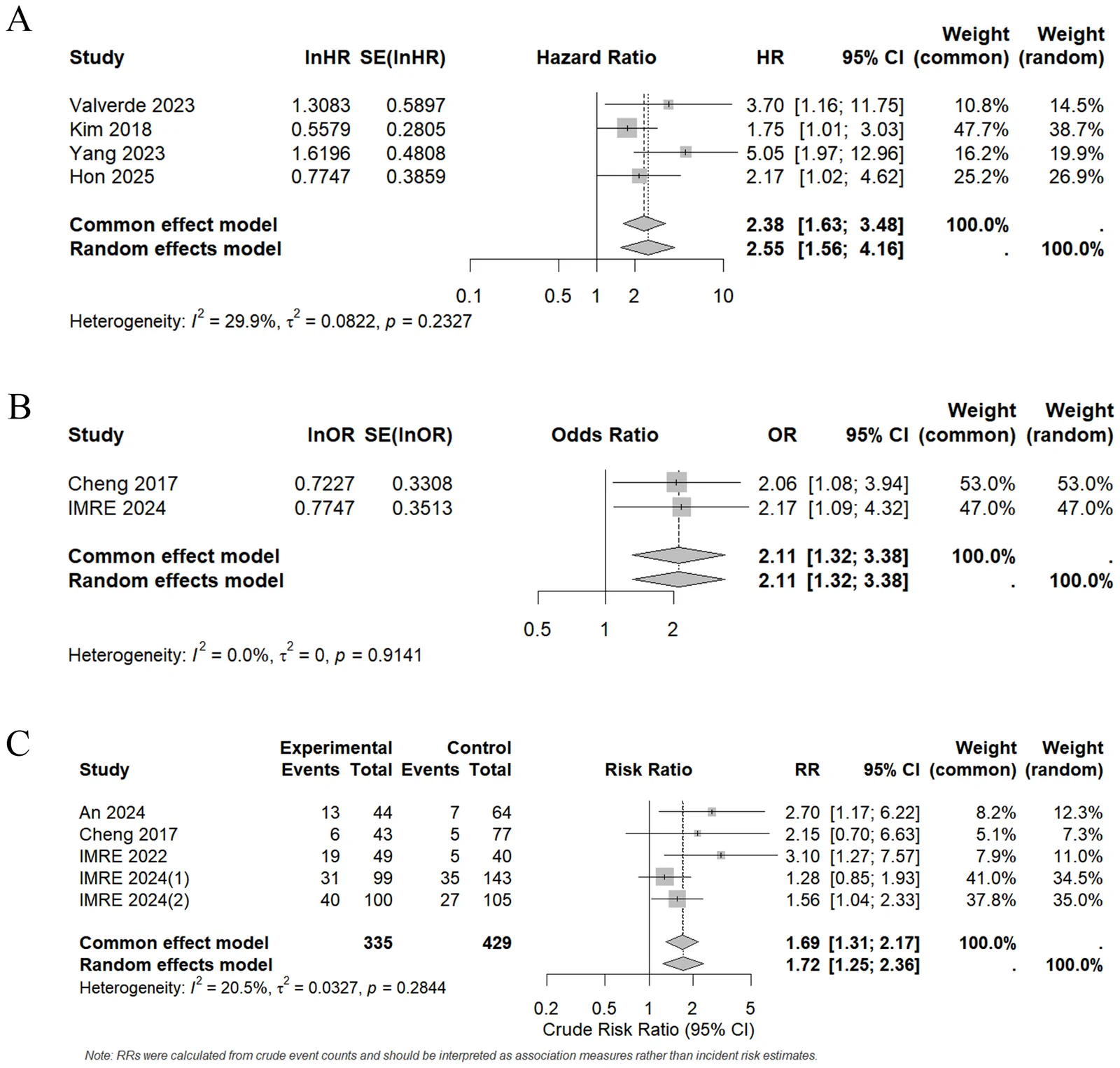
Meta-analysis results.

Four observational studies (3 prospective and 1 retrospective cohort) comprising 535 individuals were included in the meta-analysis. Given the low heterogeneity across the included studies (I² = 29.9%, P = 0.2327), a fixed-effect model was applied. The pooled HR for all-cause mortality in patients with DF and sarcopenia or low HGS was 2.38 (95% CI: 1.63–3.48; **Figure 2A**). Collectively, these results indicate that sarcopenia or reduced muscle strength is significantly associated with an increased risk of mortality in patients with DF. Leave-one-out sensitivity analysis yielded pooled HRs ranging from 2.05 to 3.15, with all 95% CIs excluding 1, indicating robust results (**Supplementary Figure 1**).

##### 3.3.1.2 Sarcopenia and amputation rate

Two investigations involving 325 individuals were pooled. Heterogeneity testing revealed I² = 0.0% and P = 0.9141, indicating no substantial heterogeneity. Therefore, a fixed-effects model was applied. The results suggested that DF patients with comorbid sarcopenia and muscle atrophy had an association with amputation (OR = 2.11, 95% CI: 1.32-3.38; **Figure 2B**).

#### 3.3.2 Secondary outcomes

##### 3.3.2.1 Sarcopenia and ulcer severity

Nine studies involving 1,202 participants reported ulcer severity. The Wagner classification was used in seven studies, while one study used the SINBAD score and one used composite severity indicators.

Five studies involving 764 participants were included in the quantitative synthesis of severe ulcers, defined as Wagner grade 4–5. Heterogeneity was low (I² = 20.5%, P = 0.2844), and a fixed-effect model was applied. The pooled crude RR suggested that sarcopenia and muscle attenuation were associated with a higher proportion of severe ulcers (RR = 1.69, 95% CI: 1.31–2.17; **Figure 2C**). Leave-one-out sensitivity analysis showed that the association remained statistically significant after excluding each study in turn, with pooled RRs ranging from 1.57 to 1.97 (**Supplementary Figure 2**).

**Figure 3:**
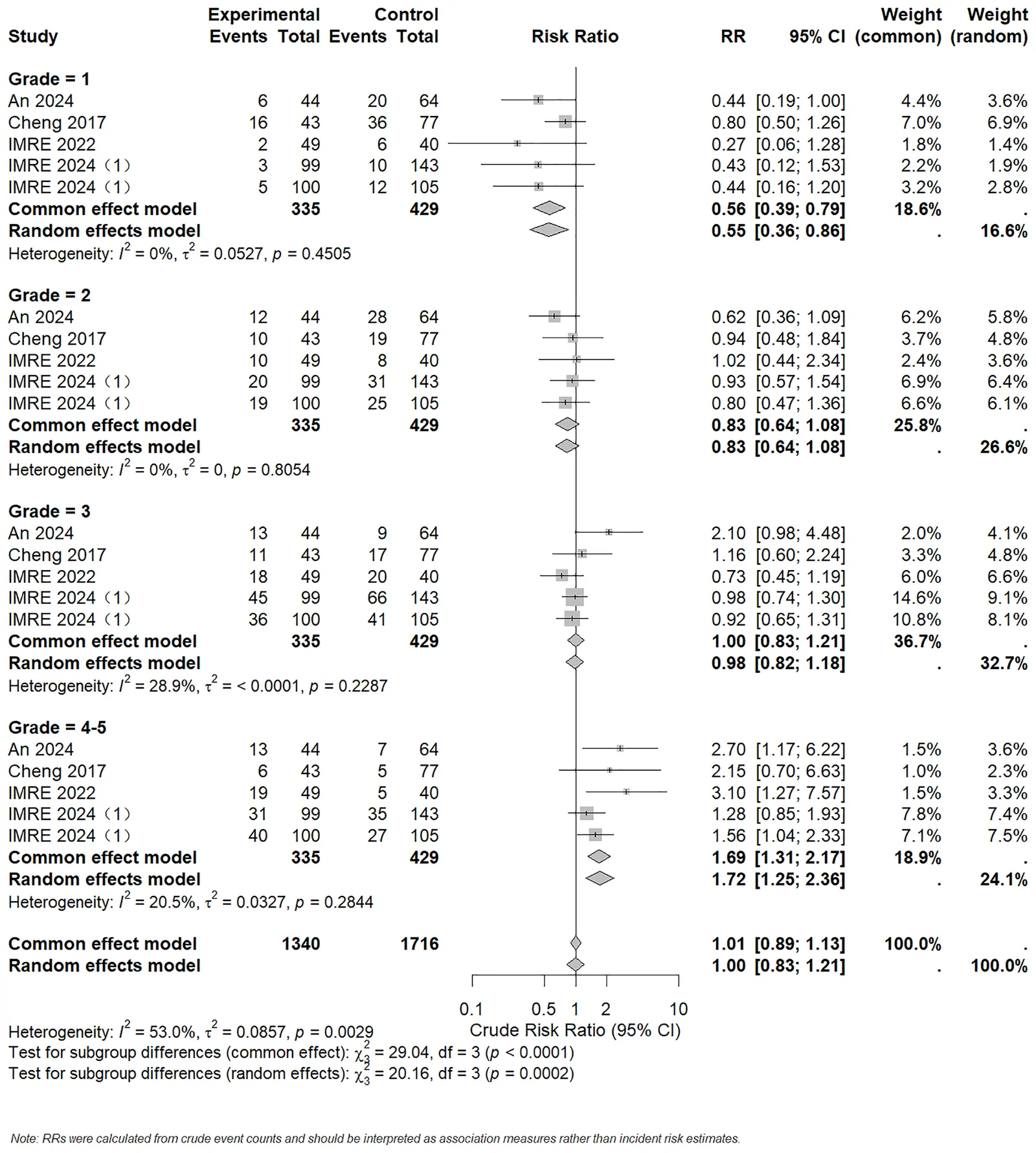
Subgroup analysis by ulcer severity.

A grade-specific analysis based on Wagner classification further suggested a possible severity gradient. Sarcopenia and muscle attenuation were associated with a lower proportion of Wagner grade 1 ulcers and a higher proportion of Wagner grade 4–5 ulcers, whereas no statistically significant associations were observed for Wagner grade 2 or grade 3 ulcers (**Figure 3**). Because the contributing studies were predominantly cross-sectional and used heterogeneous definitions of sarcopenia, these findings should be interpreted as associations with baseline ulcer severity rather than evidence of a directional prognostic effect.

#### 3.3.3 Publication bias

Funnel plots were examined to assess publication bias for two outcomes: the ulcer severity subgroups (grades 1–5, each with 5 studies) and mortality (4 studies). The funnel plots for both outcomes showed asymmetry. Each synthesis included only 4 or 5 studies. Therefore, publication bias and small-study effects could not be meaningfully assessed, and Egger’s regression tests were not performed. Funnel plots were presented for descriptive purposes only and should not be interpreted as evidence for or against publication bias (**Supplementary Figures 3**–**7**).

### 3.4 Narrative synthesis

#### 3.4.1 Sarcopenia and amputation rate

Lee et al. (18) conducted a retrospective case-control study of 351 hospitalized patients with DFU to evaluate the association between the sarcopenia index (SI) and amputation. Mean SI values did not differ significantly between the amputation and non-amputation groups (99.9 ± 95.5 vs. 101.7 ± 101.5; mean difference −1.8, P = 0.861), indicating no significant association between SI and amputation. However, given the single-center retrospective design and lack of confounder adjustment, the evidence was limited, and the predictive value of SI for DFU-related amputation required validation in prospective studies.

#### 3.4.2 Sarcopenia and wound healing rate

Two studies reported wound-healing outcomes, but quantitative pooling was not performed because of differences in exposure definitions, study design, and effect measures. He et al. (16) (retrospective cohort, SMI-defined, unadjusted) reported lower healing rates (81.82% vs. 93.02%, p = 0.046) and longer healing time (2.45 vs. 1.25 months, p = 0.039) in the sarcopenia group. Hon et al. (15) (prospective cohort, adjusted) found that high HGS was associated with the odds of healing (OR = 3.83, 95% CI: 1.35-10.92), and that per 1-kg increase in HGS, the subdistribution HR for healing was 1.04 (95% CI: 1.001–1.07), whereas muscle mass (PMI) showed no significant association. In the prospective study by Hon et al., higher HGS was associated with improved healing, though this finding is based on limited evidence and requires confirmation.

#### 3.4.3 Sarcopenia and peripheral neuropathy

Due to significant clinical heterogeneity, meta-analysis was not feasible; therefore, a narrative synthesis was presented. Yang et al. reported that DF was independently associated with sarcopenia after multivariable adjustment for age, hypertension, hemoglobin, uric acid, triglycerides, alanine aminotransferase, and diabetic peripheral neuropathy (adjusted OR = 2.138, 95% CI: 1.330–3.438).

#### 3.4.4 Sarcopenia and polyvascular disease

Two investigations jointly suggested that the characteristics for the occurrence of sarcopenia in individuals with DF may correlate with vascular disease. Lin He et al.’s cross-sectional investigation (29), analyzing 273 DF individuals with Wagner grade II ulcers, suggested that advanced age, prolonged disease duration, poor glycemic control, and severe vascular and neurological impairment might be factors associated with sarcopenia. Ni Xia et al.’s research (30) further suggested that sarcopenia might be a factor for comorbid polyvascular disease in individuals with DF (odds ratio [OR] = 3.230, 95% CI: 1.069-9.758), although this finding should be interpreted cautiously due to the cross-sectional design.

#### 3.4.5 Sarcopenia and malnutrition

A prospective observational study targeting individuals with DF aged ≥ 65 years (n = 45) (24) suggested that malnutrition and sarcopenia appeared to have a high prevalence in this population and might be relevant factors affecting prognoses in older individuals with DF.

#### 3.4.6 Sarcopenia and wound pH

Wang Hui et al.’s investigation (25), analyzing 71 individuals with DF, suggested that wound pH and SINBAD score may show a certain correlation with sarcopenia. Logistic regression suggested that elevated wound pH (OR = 29.126) and SINBAD score (OR = 5.271) might be factors associated with sarcopenia (P < 0.05). ROC curve analysis further suggested that the combination of these two indicators appeared to offer favorable diagnostic performance (area under the curve = 0.933), although this finding warrants confirmation in larger cohorts.

## 4 Discussion

### 4.1 Principal findings

This systematic review and meta-analysis encompassed 18 observational investigations and systematically examined the association of sarcopenia and muscle attenuation with clinical outcomes in individuals with DF. The findings suggested that DF individuals with comorbid sarcopenia or muscle attenuation might be associated with an elevated risk of all-cause mortality and increased associations with amputation and severe ulcers. Overall, current evidence tentatively suggests that sarcopenia and muscle attenuation may be associated with adverse outcomes in individuals with DF. Nevertheless, these results should be cautiously interpreted in light of the observational study designs, heterogeneity in exposure definitions, and outcome variability, and should be considered hypothesis-generating.

The term ‘risk’ applies to mortality outcomes derived from cohort studies. All other findings reflect associations based on a mix of cohort and cross-sectional data and should not be interpreted as causal.

Four investigations (including 3 prospective and 1 retrospective) have suggested that sarcopenia may be associated with the risk of death in individuals with DF. Prior cohort studies, irrespective of employing low HGS, low muscle mass, or a composite diagnosis of sarcopenia as the exposure indicator, largely reported relatively worse trends of survival prognosis (12–15). The pooled estimate from the current meta-analysis is broadly consistent with the aforementioned evidence, and the summary effect size suggests that sarcopenia might be associated with a certain degree of elevated risk of death in individuals with DF (HR = 2.38, 95% CI: 1.63-3.48). Notably, the influence of sarcopenia on the risk of death may not be confined solely to functional loss in musculature; rather, it appears to be a composite marker of systemic frailty, malnutrition, sustained inflammatory burden, and disease severity. Sarcopenic individuals often also have inadequate intake of protein, systemic low-grade inflammation, and compromised immune function—factors that may collectively contribute to the elevated risk of death through synergistic interactions. Hence, the value of sarcopenia in prognostic assessment for DF may stem not only from its indication of muscular functional decline but also from its possible role as a multidimensional warning indicator of adverse health status.

Sarcopenia or muscle attenuation may be associated with a higher likelihood of amputation. Kim et al.’s investigation (13) suggested that when amputation became necessary, sarcopenic individuals might tend to undergo more invasive major amputations, with a proportion of 57.1% compared with 35.3% in the non-sarcopenic group. He et al.’s research (15) reported that sarcopenic individuals might experience prolonged hospitalization and wound healing time relative to their non-sarcopenic counterparts (P < 0.05). Concerning clinical efficacy, the 1-year limb salvage rate was noted as 95.35% in the non-sarcopenic group versus 84.85% in the sarcopenic group, with a significant between-group difference (P < 0.05), suggesting that the former might enjoy a more favorable prognosis. Imre et al.’s investigation (17) found that the group of low HGS appeared to have a higher rate of major amputation (21% vs. 5%), and the overall association with undergoing any type of amputation also tended to be elevated (RR = 2.87, 95% CI: 1.60-5.23, P < 0.0001). The pooled findings suggest an association between impaired muscle health and amputation; however, whether muscle-related measures provide independent or incremental prognostic information requires further investigation.

Regarding ulcer severity, Haspolat et al. (32) reported that HGS in individuals with DFU may be inversely associated with ulcer severity. Karakousis et al. (33) suggested that low HGS might be associated with the occurrence of DFU. Among older individuals with concurrent type 2 diabetes and DF, those with low HGS appeared to have higher proportions of Wagner grades 4 and 5 (27). Prior investigations, from the perspectives of sarcopenia diagnosis or indicators for muscle function such as HGS, have suggested a possible link with greater ulcer severity. The present research further suggested that DF individuals with comorbid sarcopenia and muscle attenuation were associated with a higher proportion of severe ulcers (Wagner grades 4-5) (RR = 1.69, 95% CI: 1.31-2.17), although this should be interpreted cautiously given the predominantly cross-sectional design of the included studies.

### 4.2 Interpretation and possible mechanisms

The mechanisms linking sarcopenia and muscle attenuation to adverse outcomes of DF are likely to be multidimensional. First, loss of skeletal muscle mass and strength may, to some extent, compromise lower-extremity function in patients, leading to gait instability, impaired balance, and aberrant distribution of plantar pressure (4). This may, in turn, contribute to foot injury and the formation of ulcers. Second, sarcopenia and diabetic microvascular complications may interact reciprocally. The hyperglycemic state in diabetes may impair blood supply to muscle tissue, while microcirculatory disturbances commonly seen in sarcopenia may further affect lower-limb perfusion, potentially contributing to an ischemic and hypoxic local microenvironment. Such a microenvironment may, to a certain degree, influence tissue repair and wound healing (10). Furthermore, sarcopenia frequently co-occurs with inflammation, malnutrition, insulin resistance, and activity restriction—factors that may collectively play a role in exacerbating adverse outcomes of DF. These mechanisms may jointly contribute to the observed associations with adverse outcomes of DF in sarcopenic individuals, although present understanding remains largely based on inference from prior clinical and pathophysiological investigations.

Sarcopenia is not a localized muscle disorder but a systemic pathological state intertwined with multiple chronic comorbidities (34). In patients with diabetic foot syndrome (DFS), its presence signals a cumulative burden of long-standing diabetes, peripheral arterial disease (a key cause of lower-limb ischemia), diabetic peripheral neuropathy (a core mechanism of foot sensory loss), chronic kidney disease, and malnutrition (35–40). These comorbidities are independently associated with amputation, prolonged hospitalization, and increased all-cause mortality (41, 42).

Protein-energy malnutrition and chronic low-grade inflammation—highly prevalent in sarcopenic patients—may impair immune function and tissue repair, thereby delaying wound healing and increasing infection risk (43–45). This multisystem decline may partially mediate the association between sarcopenia and worse DFS outcomes.

Sarcopenia and other measures of impaired muscle health may reflect reduced physiological reserve and a greater burden of comorbidity. Identification of impaired muscle health may prompt further assessment of nutritional status, functional capacity, and comorbidity burden. However, whether such assessment improves clinical outcomes or risk stratification in DF remains uncertain. Prospective studies are needed to determine whether sarcopenia serves as a biomarker of multimorbidity or a modifiable driver of adverse outcomes in DFS.

### 4.3 Potential clinical relevance and research implications

HGS is a simple and feasible measure of muscle strength and may be used, within established consensus frameworks, to identify probable sarcopenia. However, consensus handgrip-strength thresholds were not developed or validated specifically for patients with DF and should not currently be interpreted as prognostic thresholds for mortality, amputation, or wound healing in this population.

The available observational evidence suggests that lower HGS and other indicators of impaired muscle health are associated with adverse clinical status and outcomes. Nevertheless, it remains uncertain whether these measures provide prognostic information beyond established factors such as age, ulcer severity, infection, peripheral arterial disease, renal dysfunction, and nutritional status.

Assessment of muscle health may prompt broader evaluation of nutritional status, physical function, and comorbidity burden. Further prospective studies should determine the independent and incremental prognostic value of muscle-related measures, identify appropriate DF-specific thresholds, and evaluate whether screening or targeted interventions improve patient outcomes.

### 4.4 Methodological considerations

The evidence base is primarily observational, with most included studies being cross-sectional in design, which precludes causal inference and limits assessment of temporal relationships between sarcopenia and DF outcomes. The diagnostic criteria for sarcopenia also varied considerably across studies, with some using AWGS criteria (46) and others applying EWGSOP/EWGSOP2 standards (45, 47); several studies did not adopt complete consensus definitions but relied on single indicators—such as grip strength or muscle mass alone—as surrogate exposures (46). This methodological diversity reinforces the need for caution when interpreting pooled estimates derived from heterogeneous exposure definitions (48, 49). Moreover, the pooled estimate for ulcer severity was derived from unadjusted data, as adjusted estimates reported in primary studies for this outcome varied considerably in their adjustment sets and outcome definitions, precluding meaningful pooling. In contrast, the pooled estimates for mortality and amputation were based on adjusted data; consequently, summary effects may be influenced by residual confounding. Key confounders were not consistently adjusted for across studies, and the likely direction of residual confounding is toward overestimation, as patients with sarcopenia tend to be older, have longer diabetes duration, and present with more comorbidities, all of which independently predict worse DF outcomes. Adjustment for factors on the causal pathway could lead to underestimation. Thus, the true associations may be more modest than suggested by the current pooled estimates.

### 4.5 Emerging metabolic context

In addition, sarcopenia has recently attracted increasing attention in metabolic medicine, particularly in the context of incretin-based weight-loss therapies, where preservation of lean body mass has become an important clinical concern (50). Emerging muscle-sparing strategies, including myostatin/activin pathway inhibitors such as bimagrumab, further highlight the clinical relevance of muscle health (51–54). For patients with DFS, who often present with frailty, malnutrition, chronic inflammation, and vascular disease, careful monitoring of body composition may be particularly important in future metabolic and multidisciplinary care pathways (50, 55).

### 4.6 Strengths and limitations

To our knowledge, the present investigation represents one of the earlier systematic reviews and meta-analyses to synthesize available evidence on the association of sarcopenia and muscle attenuation with adverse outcomes in individuals with DF, which may offer some systematic evidence-based support for understanding the relationship between these two clinical problems. By incorporating multiple outcomes—death, amputation, ulcer severity—this investigation may afford a broader perspective on the risk profile of individuals with DF.

Nevertheless, several limitations should be acknowledged. First, the number of included studies was relatively limited, with the majority being cross-sectional; thus, temporal sequences and causal relationships between sarcopenia and adverse DF outcomes cannot be established with certainty. Second, confounding control was inadequate in several included studies, and many studies failed to adjust for key confounders such as age, sex, BMI, diabetes duration, vascular disease, infection severity, and baseline functional status. While pooled estimates for mortality and amputation incorporated adjusted effect sizes, the estimate for ulcer severity was based solely on unadjusted data due to heterogeneity in adjustment strategies across studies. This residual confounding may have led to overestimation of the observed associations, particularly for ulcer severity. The findings should therefore be interpreted as hypothesis-generating rather than confirmatory. Third, sarcopenia was not uniformly defined across studies. Some employed rigorous diagnostic criteria, while others utilized low muscle mass, low muscle density, or low HGS as surrogate exposures, potentially affecting comparability. Fourth, outcome definitions and measurement modalities varied. For example, amputation type, healing-related outcomes, ulcer severity scoring, and peripheral neuropathy assessment were not fully standardized. Fifth, certain outcomes—particularly peripheral neuropathy and healing-related endpoints—included too few studies to provide adequate statistical power. Sixth, formal tests for publication bias (Egger’s regression) and meta-regression could not be performed due to the insufficient number of studies (< 10 per outcome). The small number of studies also restricted the comprehensive exploration of heterogeneity sources beyond subgroup analyses.

## 5 Conclusions

Sarcopenia and muscle attenuation may be associated with higher all-cause mortality, amputation, and severe ulcer status in individuals with DF. However, given that the available evidence is mainly observational and heterogeneous, and the estimate of ulcer severity is based on unadjusted data, these findings should be considered hypothesis-generating. Further prospective studies with standardized sarcopenia assessment and adequate confounding adjustment are needed.

## Data Availability

The original contributions presented in the study are included in the article/**Supplementary Material**. Further inquiries can be directed to the corresponding authors.
