# Supplementary material for: Association of sarcopenia and muscle attenuation with clinical outcomes in patients with diabetic foot: a systematic review and meta-analysis
Source: Front Endocrinol (Lausanne). 2026 Aug 21;17:1894356. doi: 10.3389/fendo.2026.1894356 (PMC13542880; doi:10.3389/fendo.2026.1894356)
Supplement: Supplementary file 1 [file Supplementaryfile1.docx]

**Supplementary Table S1 PRISMA 2020 Checklist**

| **Section and Topic** | **Item #** | **Checklist item** | **Location where item is reported** |
| --- | --- | --- | --- |
| **TITLE** | | |  |
| Title | 1 | Identify the report as a systematic review. | Title page |
| **ABSTRACT** | | |  |
| Abstract | 2 | See the PRISMA 2020 for Abstracts checklist. | Abstract |
| **INTRODUCTION** | | |  |
| Rationale | 3 | Describe the rationale for the review in the context of existing knowledge. | 1.Background,  paragraph 1-3 |
| Objectives | 4 | Provide an explicit statement of the objective(s) or question(s) the review addresses. | 1. Background,  final paragraph |
| **METHODS** | | |  |
| Eligibility criteria | 5 | Specify the inclusion and exclusion criteria for the review and how studies were grouped for the syntheses. | 2.2 Study Selection  2.6 Data Synthesis |
| Information sources | 6 | Specify all databases, registers, websites, organisations, reference lists and other sources searched or consulted to identify studies. Specify the date when each source was last searched or consulted. | 2.1 Data Sources |
| Search strategy | 7 | Present the full search strategies for all databases, registers and websites, including any filters and limits used. | 2.1 Data Sources  Supplementary Table S2 |
| Selection process | 8 | Specify the methods used to decide whether a study met the inclusion criteria of the review, including how many reviewers screened each record and each report retrieved, whether they worked independently, and if applicable, details of automation tools used in the process. | 2.3 Literature screening |
| Data collection process | 9 | Specify the methods used to collect data from reports, including how many reviewers collected data from each report, whether they worked independently, any processes for obtaining or confirming data from study investigators, and if applicable, details of automation tools used in the process. | 2.5 Data Extraction |
| Data items | 10a | List and define all outcomes for which data were sought. Specify whether all results that were compatible with each outcome domain in each study were sought (e.g. for all measures, time points, analyses), and if not, the methods used to decide which results to collect. | 2.2 Study Selection  2.5 Data Extraction |
|  | 10b | List and define all other variables for which data were sought (e.g. participant and intervention characteristics, funding sources). Describe any assumptions made about any missing or unclear information. | 2.5 Data Extraction  Table 1 Note |
| Study risk of bias assessment | 11 | Specify the methods used to assess risk of bias in the included studies, including details of the tool(s) used, how many reviewers assessed each study and whether they worked independently, and if applicable, details of automation tools used in the process. | 2.4 Risk-of-Bias Assessment  Supplementary Table S5 |
| Effect measures | 12 | Specify for each outcome the effect measure(s) (e.g. risk ratio, mean difference) used in the synthesis or presentation of results. | 2.6 Data Synthesis |
| Synthesis methods | 13a | Describe the processes used to decide which studies were eligible for each synthesis (e.g. tabulating the study intervention characteristics and comparing against the planned groups for each synthesis (item #5)). | 2.2 Study Selection  2.6 Data Synthesis |
|  | 13b | Describe any methods required to prepare the data for presentation or synthesis, such as handling of missing summary statistics, or data conversions. | 2.5 Data Extraction  Table 1 Note |
|  | 13c | Describe any methods used to tabulate or visually display results of individual studies and syntheses. | 2.6 Data Synthesis |
|  | 13d | Describe any methods used to synthesize results and provide a rationale for the choice(s). If meta-analysis was performed, describe the model(s), method(s) to identify the presence and extent of statistical heterogeneity, and software package(s) used. | 2.6 Data Synthesis |
|  | 13e | Describe any methods used to explore possible causes of heterogeneity among study results (e.g. subgroup analysis, meta-regression). | 2.6 Data Synthesis |
|  | 13f | Describe any sensitivity analyses conducted to assess robustness of the synthesized results. | 2.6 Data Synthesis  3.3.1 Primary Outcomes 3.3.2 Secondary Outcomes |
| Reporting bias assessment | 14 | Describe any methods used to assess risk of bias due to missing results in a synthesis (arising from reporting biases). | 2.6 Data Synthesis |
| Certainty assessment | 15 | Describe any methods used to assess certainty (or confidence) in the body of evidence for an outcome. | Certainty of evidence was not formally assessed. |
| **RESULTS** | | |  |
| Study selection | 16a | Describe the results of the search and selection process, from the number of records identified in the search to the number of studies included in the review, ideally using a flow diagram. | 3.1 Study Selection  Figure 1 |
|  | 16b | Cite studies that might appear to meet the inclusion criteria, but which were excluded, and explain why they were excluded. | 3.1 Study Selection |
| Study characteristics | 17 | Cite each included study and present its characteristics. | 3.2.1 Baseline Characteristics  Table 1 |
| Risk of bias in studies | 18 | Present assessments of risk of bias for each included study. | 3.2.2 Risk-of-Bias Assessment  Supplementary Table S5 |
| Results of individual studies | 19 | For all outcomes, present, for each study: (a) summary statistics for each group (where appropriate) and (b) an effect estimate and its precision (e.g. confidence/credible interval), ideally using structured tables or plots. | 3.3 Meta-analysis results  Figure 2A-C  3.4 Narrative Synthesis |
| Results of syntheses | 20a | For each synthesis, briefly summarise the characteristics and risk of bias among contributing studies. | 3.3 Meta-analysis results |
|  | 20b | Present results of all statistical syntheses conducted. If meta-analysis was done, present for each the summary estimate and its precision (e.g. confidence/credible interval) and measures of statistical heterogeneity. If comparing groups, describe the direction of the effect. | 3.3.1 Primary Outcomes 3.3.2 Secondary Outcomes  Figure 2A-C |
|  | 20c | Present results of all investigations of possible causes of heterogeneity among study results. | Not applicable. Meta-regression and formal subgroup analyses to investigate sources of heterogeneity were not performed because of the limited number of studies. |
|  | 20d | Present results of all sensitivity analyses conducted to assess the robustness of the synthesized results. | 3.3.1 Primary Outcomes 3.3.2 Secondary Outcomes  Supplementary Figures S1-S2 |
| Reporting biases | 21 | Present assessments of risk of bias due to missing results (arising from reporting biases) for each synthesis assessed. | 3.3.3 Publication bias Supplementary Figures S3-S7 |
| Certainty of evidence | 22 | Present assessments of certainty (or confidence) in the body of evidence for each outcome assessed. | No formal certainty-of-evidence assessment was performed. |
| **DISCUSSION** | | |  |
| Discussion | 23a | Provide a general interpretation of the results in the context of other evidence. | 4. Discussion |
|  | 23b | Discuss any limitations of the evidence included in the review. | 4.6 Strengths and Limitations |
|  | 23c | Discuss any limitations of the review processes used. | 4.6 Strengths and Limitations |
|  | 23d | Discuss implications of the results for practice, policy, and future research. | 4.2 Interpretation and possible mechanisms  4.3 Potential clinical relevance and research implications  5. Conclusions |
| **OTHER INFORMATION** | | |  |
| Registration and protocol | 24a | Provide registration information for the review, including register name and registration number, or state that the review was not registered. | 2. Methods  Abstract |
|  | 24b | Indicate where the review protocol can be accessed, or state that a protocol was not prepared. | Yes, CRD420251247873 |
|  | 24c | Describe and explain any amendments to information provided at registration or in the protocol. | Not applicable |
| Support | 25 | Describe sources of financial or non-financial support for the review, and the role of the funders or sponsors in the review. | Funding Sources  Abstract |
| Competing interests | 26 | Declare any competing interests of review authors. | Conflicts of Interest |
| Availability of data, code and other materials | 27 | Report which of the following are publicly available and where they can be found: template data collection forms; data extracted from included studies; data used for all analyses; analytic code; any other materials used in the review. | Data statement  Supplementary materials |

*From:* Page MJ, McKenzie JE, Bossuyt PM, Boutron I, Hoffmann TC, Mulrow CD, et al. The PRISMA 2020 statement: an updated guideline for reporting systematic reviews. BMJ 2021;372:n71. doi: 10.1136/bmj.n71

Supplementary Table S2 Search Strategy

| **Database** | **Search details** | **Results** | **Date** |
| --- | --- | --- | --- |
| PubMed | ((diabeticfoot[MeSHTerms])OR(DiabeticFoot[Title/Abstract]ORDiabeticFeet[Title/Abstract]ORdiabeticfootdisease[Title/Abstract]ORdiabeticfootsyndrome[Title/Abstract]ORFeetDiabetic[Title/Abstract]ORFootDiabetic[Title/Abstract]ORFootUlcerDiabetic[Title/Abstract]))AND(("Sarcopenia"[Mesh])OR(Sarcopenia[Title/Abstract]ORageingrelatedmuscleatrophy[Title/Abstract]ORagerelatedmuscleatrophy[Title/Abstract]ORagerelatedmuscledecline[Title/Abstract]ORagerelatedmusculardecline[Title/Abstract]ORagerelatedmusculardegeneration[Title/Abstract]ORgeriatricmuscularatrophy[Title/Abstract]ORSarcopenias[Title/Abstract]ORmuscle[Title/Abstract]ORmuscular[Title/Abstract])) | 356 | 2025/12/3 |
| Embase | ('diabeticfoot':ab,tiOR'diabeticfeet':ab,tiOR'diabeticfootdisease':ab,tiOR'diabeticfootsyndrome':ab,tiOR'feetdiabetic':ab,tiOR'footdiabetic':ab,tiOR'footulcerdiabetic':ab,ti)AND(sarcopenia:ab,tiOR'ageingrelatedmuscleatrophy':ab,tiOR'agerelatedmuscleatrophy':ab,tiOR'agerelatedmuscledecline':ab,tiOR'agerelatedmusculardecline':ab,tiOR'agerelatedmusculardegeneration':ab,tiOR'geriatricmuscularatrophy':ab,tiORsarcopenias:ab,tiORmuscle:ab,tiORmuscular:ab,ti) | 589 | 2025/12/3 |
| Cochrane | (MeSHdescriptor:[DiabeticFoot]explodealltrees)OR(DiabeticFootORDiabeticFeetORdiabeticfootdiseaseORdiabeticfootsyndromeORFeetDiabeticORFootDiabeticORFootUlcerDiabetic):ti,ab,kwAND(MeSHdescriptor:[Sarcopenia]explodealltrees)OR(SarcopeniaORageingrelatedmuscleatrophyORagerelatedmuscleatrophyORagerelatedmuscledeclineORagerelatedmusculardeclineORagerelatedmusculardegenerationORgeriatricmuscularatrophyORSarcopeniasORmuscleORmuscular):ti,ab,kw | 245 | 2025/12/3 |
| Web of Science | "DiabeticFoot"OR"DiabeticFeet"OR"diabeticfootdisease"OR"diabeticfootsyndrome"OR"FeetDiabetic"OR"FootDiabetic"OR"FootUlcerDiabetic" (Topic) and "Sarcopenia"OR"ageingrelatedmuscleatrophy"OR"agerelatedmuscleatrophy"OR"agerelatedmuscledecline"OR"agerelatedmusculardecline"OR"agerelatedmusculardegeneration"OR"geriatricmuscularatrophy"OR"Sarcopenias"OR"muscle"OR"muscular" (Topic) | 415 | 2025/12/3 |
| 中国知网 | (篇关摘:糖尿病足+diabetesfoot+diabeticfeet+diabeticfoot+糖尿病肢端坏疽+糖尿病坏疽(精确))AND(篇关摘:骨骼肌减少症+sarcopenia+肌肉減少症+少肌症+肌少症(精确)) | 30 | 2025/12/3 |
| 万方 | 题名或关键词:(糖尿病足OR"diabetesfoot"OR"diabeticfeet"OR"diabeticfoot"OR糖尿病肢端坏疽OR糖尿病坏疽)and题名或关键词:(骨骼肌减少症ORsarcopeniaOR肌肉减少症OR少肌症OR肌少症) | 26 | 2025/12/3 |
| 维普 | ((((((题名或关键词=糖尿病足OR题名或关键词=diabetesfoot)OR题名或关键词=diabeticfeet)OR题名或关键词=diabeticfoot)OR题名或关键词=糖尿病肢端坏疽)OR题名或关键词=糖尿病坏疽)AND(((((题名或关键词=骨骼肌减少症OR题名或关键词=sarcopenia)OR题名或关键词=肌肉减少症)OR题名或关键词=少肌症)OR题名或关键词=肌少症)OR题名或关键词=肌肉)) | 62 | 2025/12/3 |
| Sinomed | ("糖尿病足"[常用字段:智能]OR""diabetesfoot""[常用字段:智能]OR""diabeticfeet""[常用字段:智能]OR""diabeticfoot""[常用字段:智能]OR"糖尿病肢端坏疽"[常用字段:智能]OR"糖尿病坏疽"[常用字段:智能])AND("骨骼肌减少症"[常用字段:智能]OR"sarcopenia"[常用字段:智能]OR"肌肉减少症"[常用字段:智能]OR"少肌症"[常用字段:智能]OR"肌少症"[常用字段:智能]) | 44 | 2025/12/3 |

Supplementary Table S3 Diagnostic Criteria for Sarcopenia

| **NO.** | **First Author** | **Year** | **Study design** | **Country** | **Diagnostic criteria for sarcopenia** | **Exposure category** |
| --- | --- | --- | --- | --- | --- | --- |
| 1 | An | 2024 | Cross-sectional study | China | Based on the AWGS 2019 diagnostic consensus: ASMI (DXA) - men: <7.0 kg/m², women: <5.4 kg/m²; grip strength - men: <28 kg, women: <18 kg; 5 sit-to-stand tests ≥12 seconds. Diagnostic pathway: muscle mass reduction + (strength or function decline). | Consensus-defined sarcopenia |
| 2 | Yang | 2020 | Cross-sectional study | China | Baumgartner diagnostic criteria (1988): ALMI (DXA) - male: <7.01 kg/m², female: <5.42 kg/m² | Isolated low muscle quantity/mass |
| 3 | He | 2020 | Retrospective Cohort | China | SMI (CTatT12) - Male: <42.6 cm²/m², Female: <30.6 cm²/m² | Isolated low muscle quantity/mass |
| 4 | Valverde | 2023 | Prospective Cohort | Spain | The average of three grip strength measurements | Isolated low muscle strength |
| 5 | Harmand | 2023 | Retrospective Cohort | Spain | Grip strength in reference to the values of adults in the Teruel region of Spain (Nuevastablasdefuerzadelamano) | Isolated low muscle strength |
| 6 | Lee | 2020 | Retrospective case-control | Korea | Sarcopenia Index (SI) - Formula: Serum creatinine value / Cystatin C value (diagnostic criteria not specified) | Biochemical surrogate index |
| 7 | Kim | 2018 | Retrospective Cohort | Korea | L3-SMI (CT): Male: <52.4 cm²/m², Female: <38.5 cm²/m² | Isolated low muscle quantity/mass |
| 8 | Yang | 2023 | Prospective Cohort | China | Baumgartner diagnostic criteria: ALMI (DXA) - Male: <7.01 kg/m², Female: <5.42 kg/m² | Isolated low muscle quantity/mass |
| 9 | Cheng | 2017 | Cross-sectional study | China | SMI(DXA)-male: <7.0kg/m², female: <5.4kg/m² | Isolated low muscle quantity/mass |
| 10 | Wang | 2025 | Cross-sectional study | China | Chinese population-specific SMI+EWGSOP general standards: SMI (CTatL3) - Male: ≤40.8cm²/m², Female: ≤39.4cm²/m²; Grip strength (Baseline grip strength meter) - Male: <27kg, Female: <16kg | Non-consensus composite or mixed definition |
| 11 | Hon | 2025 | Prospective Cohort | Australia | PMI (CTatL3) - reduced by gender Q1 (lowest quartile);  Grip strength (TTM hand strength meter) - male: <27kg, female: <16kg (EWGSOP2 standard) | Non-consensus composite or mixed definition |
| 12 | He Lin | 2017 | Cross-sectional study | China | Asian Sarcopenia Working Group (AWGS) Recommendations: ASMI (BIA, TANITAMC180) - men: <7.0 kg/m², women: <5.7 kg/m²; grip strength (Jamar Grip Analyzer) - men: <26 kg, women: <18 kg; SPPB (4m walking speed <0.8 m/s) | Consensus-defined sarcopenia |
| 13 | Xia Ni | 2023 | Cross-sectional study | China | Baumgartner diagnostic criteria: ASMI (DXA) - Male: <7.01 kg/m², Female: <5.42 kg/m² | Isolated low muscle quantity/mass |
| 14 | Jie Bai | 2023 | Cross-sectional study | China | Comprehensive assessment (any abnormal indicator): Skeletal muscle mass index - males: <7.0 kg/m², females: <5.7 kg/m²; Grip strength - males: <26 kg, females: <18 kg; 4m walking speed <0.8 m/s | Non-consensus composite or mixed definition |
| 15 | IMRE | 2022 | Cross-sectional study | Turkey | Grip strength (Smedley digital muscle strength meter) - Men: <27kg, Women: <16kg | Isolated low muscle strength |
| 16 | IMRE | 2024 | Cross-sectional study | Turkey | Grip strength (Smedley digital muscle strength meter) - Men: <27kg, Women: <16kg | Isolated low muscle strength |
| 17 | IMRE | 2024 | Prospective Cohort | Turkey | Grip strength (Smedley digital myometry meter) - no specific threshold | Isolated low muscle strength |
| 18 | Jung | 2023 | Retrospective Cohort | Korea | Cross-sectional area (CSA) and attenuation (ATT) to estimate muscle volume and density | Continuous or imaging-derived muscle quantity measure; diagnostic cut-off not reported |

Supplementary Table S4 Outcome-by-study matrix for the quantitative syntheses

| **Study** | **Mortality, adjusted HR** | **Amputation, adjusted OR** | **Severe ulcer, crude RR** |
| --- | --- | --- | --- |
| An et al., 2024 | — | — | √ |
| Valverde et al., 2023 | √ | — | — |
| Kim et al., 2018 | √ | — | — |
| Yang et al., 2023 | √ | — | — |
| Cheng et al., 2017 | — | √ | √ |
| Hon et al., 2025 | √ | — | — |
| Imre et al., 2022 | — | — | √ |
| Imre et al., 2024, cross-sectional | — | — | √ |
| Imre et al., 2024, prospective cohort | — | √ | √ |
| **Number of contributing studies** | **4** | **2** | **5** |

Supplementary Table S5 Quality Assessment Results of Observational Cohort and Cross-sectional Studies by the National

| **Study** | **Q1** | **Q2** | **Q3** | **Q4** | **Q5** | **Q6** | **Q7** | **Q8** | **Q9** | **Q10** | **Q11** | **Q12** | **Q13** | **Q14** | **Outcome** |
| --- | --- | --- | --- | --- | --- | --- | --- | --- | --- | --- | --- | --- | --- | --- | --- |
| Jung 2023 | √ | √ | √ | √ | × | √ | √ | √ | √ | NA | × | NR | × | √ | Fair |
| An 2024 | √ | √ | NR | √ | × | NA | NA | √ | √ | NA | √ | NR | NA | √ | Fair |
| Yang 2020 | √ | √ | × | √ | × | NA | NA | √ | √ | NA | √ | NR | NA | √ | Fair |
| He 2020 | √ | √ | √ | √ | × | √ | √ | NR | √ | × | √ | NR | √ | × | Fair |
| Valverde 2023 | √ | √ | √ | √ | √ | √ | √ | × | √ | × | √ | NR | √ | √ | **Good** |
| Harmand 2023 | √ | √ | √ | √ | × | √ | √ | √ | √ | × | √ | NR | √ | × | Fair |
| Lee 2020 | √ | √ | √ | √ | × | √ | √ | √ | √ | × | √ | NR | NA | √ | **Good** |
| Kim 2018 | √ | √ | √ | √ | × | √ | √ | NR | √ | × | √ | NR | √ | √ | **Good** |
| Yang 2023 | √ | √ | √ | √ | × | √ | √ | NR | √ | × | √ | NR | √ | √ | **Good** |
| Cheng 2017 | √ | √ | √ | √ | × | NA | NA | √ | √ | NA | √ | NR | NA | √ | **Good** |
| Wang 2025 | √ | √ | √ | × | × | NA | NA | √ | √ | NA | √ | NR | NA | √ | Fair |
| Hon 2025 | √ | √ | √ | √ | × | √ | √ | √ | √ | × | √ | NR | √ | √ | **Good** |
| He Lin 2017 | √ | √ | × | √ | × | NA | NA | √ | √ | NA | √ | NR | NA | √ | Fair |
| Xia Ni 2023 | √ | √ | √ | × | × | NA | NA | √ | √ | NA | √ | NR | NA | √ | Fair |
| Jie Bai 2023 | √ | √ | × | √ | × | NA | NA | √ | × | NA | √ | NR | NA | √ | Fair |
| IMRE 2022 | √ | √ | NR | √ | √ | NA | NA | √ | √ | NA | √ | NR | NA | √ | Fair |
| IMRE 2024(1) | √ | √ | √ | √ | √ | NA | NA | NR | √ | NA | √ | NR | NA | √ | Fair |
| IMRE 2024(2) | √ | √ | √ | √ | √ | √ | √ | √ | √ | × | √ | NR | √ | √ | **Good** |

Heart, Lung, and Blood Institute (NHLBI)

Notes: Q1:Was the research question or objective in this paper clearly stated?

Q2:Was the study population clearly specified and defined?

Q3:Was the participation rate of eligible persons at least 50%?

Q4:Were all the subjects selected or recruited from the same or similar populations (including the same time period)? Were inclusion and exclusion criteria for being in the study prespecified and applied uniformly to all participants?

Q5:Was a sample size justification, power description, or variance and effect estimates provided?

Q6:For the analyses in this paper, were the exposure(s) of interest measured prior to the outcome(s) being measured?

Q7:Was the timeframe sufficient so that one could reasonably expect to see an association between exposure and outcome if it existed?

Q8:For exposures that can vary in amount or level, did the study examine different levels of the exposure as related to the outcome (e.g., categories of exposure, or exposure measured as continuous variable)?

Q9:Were the exposure measures (independent variables) clearly defined, valid, reliable, and implemented consistently across all study participants?

Q10:Was the exposure(s) assessed more than once over time?

Q11:Were the outcome measures (dependent variables) clearly defined, valid, reliable, and implemented consistently across all study participants?

Q12:Were the outcome assessors blinded to the exposure status of participants?

Q13:Was loss to follow-up after baseline 20% or less?

Q14:Were key potential confounding variables measured and adjusted statistically for their impact on the relationship between exposure(s) and outcome(s)

Supplementary Figure S1 Leave-one-out sensitivity analysis for mortality rate

Supplementary Figure S2 Leave-one-out sensitivity analysis for ulcer severity

Supplementary Figure S3 Funnel plot for ulcer severity (Wagner grade 1)

Supplementary Figure S4 Funnel plot for ulcer severity (Wagner grade 2)

Supplementary Figure S5 Funnel plot for ulcer severity (Wagner grade 3)

Supplementary Figure S6 Funnel plot for ulcer severity (Wagner grades 4-5)

Supplementary Figure S7 Funnel plot for mortality
